# Outcomes of temporalis muscle-based facial reanimation surgery: A systematic review and meta-analysis

**DOI:** 10.1016/j.jpra.2024.10.015

**Published:** 2024-11-01

**Authors:** Zhen Yu Wong, Frank W. de Jongh, Koen J.A.O. Ingels, Niels van Heerbeek, Sjaak Pouwels

**Affiliations:** a Department of General Surgery, Nottingham City Hospital, Nottingham, United Kingdom; b Department of Otorhinolaryngology and Head & Neck Surgery, Radboudumc, Nijmegen, The Netherlands; c Department of Surgery, Marien Hospital Herne, University Hospital of Ruhr University Bochum, Herne, NRW, Germany; d Department of Intensive Care Medicine, Elisabeth-Tweesteden Hospital Tilburg, The Netherlands

**Keywords:** Labbé procedure, Facial reanimation surgery, Facial palsy, Plastic surgery, Peripheral facial palsy

## Abstract

**Background:**

Despite the encouraging findings of temporalis muscle-based facial reanimation surgery without the need for nerve grafting, there is a need for comprehensive evaluation of the impact of temporalis-based facial reanimation surgery on key outcome measures.

**Methods:**

Comprehensive search in Medline and Embase databases were carried out up to 25 February 2023. The articles that examined facial reanimation surgery using the temporalis muscle were included in this study. Postoperative changes in smile excursion and the angle of the mouth while smiling were pooled using the DerSimonian and Laird random-effects model. Narrative synthesis was conducted for other outcomes including assessments of spontaneous smile, subjective evaluation of facial symmetry using validated rating tools, functional outcomes, aesthetic outcomes and patient-reported outcomes owing to heterogeneity in reporting of the outcomes.

**Results:**

Twenty-four studies were included in the analysis. Conflicting evidence was demonstrated regarding emotional smile outcomes and its definition. The pooled changes in smile excursion postsurgery were 7.06 mm (95% CI: 3.73-10.40, P < 0.001; I^2^ = 0%) and the angle of the mouth were 11.76° (95% CI: 8.80-14.71, P < 0.001; I^2^ = 0%). Significant improvement was reported across the validated rating scales of symmetry, functional outcomes, aesthetic outcomes and patient-reported outcomes whereas the superiority compared to other procedures remained inconclusive.

**Conclusion:**

Overall, temporalis-based facial reanimation surgery is a promising option for addressing the negative effects of facial nerve paralysis on the patients’ quality of life. This study highlights the uncertainty surrounding the technique and need for further studies.

## Introduction

Facial nerve paralysis is considered as one of the most complex and potentially debilitating conditions of the human face. This disease affects functional, aesthetic and psychosocial aspects and can lead to social isolation, psychological distress and more severe forms of psychiatric conditions such as depression and anxiety disorder. Moreover, facial nerve paralysis can significantly impact the quality of life of patients.^1^^,^^2^ Nowadays, there are multiple (surgical) options to restore facial expression, including nerve transfers, muscle transpositions and free muscle transfers. As the human face is essential in interpersonal communication and social interaction, the role of facial expressions is of utmost importance.^3^ Since the first documented nerve transfer for the restoration of facial (nerve) function in 1800, surgeons have challenged the boundaries of facial reanimation surgery. This included significantly evolved surgical techniques, aimed at restoring a “spontaneous” smile and improving the functional and aesthetic outcomes, while minimizing the potential “complications” of autologous nerve grafting and synkinesis. These advancements encompassing nerve transfers (involving the redirection of healthy nerves), muscle transpositions (involving the relocation of muscles within the body) and free muscle transfers (comprising the detachment and reattachment of muscles to new blood vessels and nerves using microsurgical techniques) comprise static and dynamic interventions, with a primary emphasis on early surgical intervention to mitigate the deleterious effects of prolonged muscle denervation.^4^

Nerve transfers are frequently used in facial reanimation surgery and the decision for using such techniques is dependent on the presence (or absence) of the functionality of the facial muscles. However, the efficacy of alternative option such as temporalis-based facial reanimation surgery including temporalis muscle/tendon transfer (TTT) and lengthening temporalis myoplasty (LTM) remains poorly investigated.^5^ Despite a previous meta-analysis discussing the efficacy of LTM, the findings were limited by the scarcity of the included studies, weak evidence, absence of meta-analysis and primarily relying on subjective evaluation outcomes. Consequently, there is a need for a more comprehensive review to provide a more in-depth understanding of the efficacy of temporalis surgery in facial paralysis.^6^ The effectiveness of temporalis-based facial reanimation surgery as a means of generating spontaneous smiles in patients, without the need for nerve grafting, is a subject of interest and inquiry within the field of reconstructive surgery. Despite the encouraging findings, there remains a need for comprehensive evaluation of the impact of temporalis-based facial reanimation surgery on key outcome measures, such as smile excursion, functional outcomes, symmetry, aesthetic outcomes and patient-reported outcomes. The goal of this review was to provide a comprehensive overview of the effectiveness of temporalis-based facial reanimation surgery in facial paralysis.

## Materials and methods

A systematic literature search was conducted. The Population, Intervention, Comparison, Outcome (Therapy) (PICO(T)) method was used to define our search strategy. The population of interest was patients who underwent TTT and LTM. Interventions were TTT and LTM regardless of the variation in technique and our outcomes of interest consisted of the following:

1. Outcomes in spontaneous smile
2. Postoperative changes in smile excursion
3. Postoperative changes in the angle of the mouth while smiling
4. Subjective measurement of facial symmetry based on validated rating tools
5. Objective measurement of the functional outcomes
6. Aesthetic outcomes
7. Patient-reported outcomes

### Search strategy and data sources

To conduct a comprehensive literature search, the following databases were consulted: Medline and Embase. Searches were conducted from the earliest date available in each database up to 15 February 2022, using permutations of keywords “Labbé procedure” and “temporalis muscle transfer.” Gray literature was not included in the search. All articles in English, Dutch and German were included. The search results were collected using Endnote version 20 for MacIntosh and screened by authors ZYW and FdJ. Preliminary screening of titles and abstracts followed by a full-text review of the selected studies were carried out based on the established inclusion criteria. Any conflicts were resolved by consulting the senior author SP. Relevant studies were identified by examining the reference lists of the included articles.

### Inclusion criteria

To ensure a rigorous and comprehensive review, several study designs were considered for inclusion in this analysis, including case series, retrospective and prospective cohort studies and randomized controlled trials. However, to maintain a focus on high-quality evidence and avoid potential bias, certain types of articles, such as case reports, editorials, systematic reviews, meta-analyses and commentaries, were excluded from consideration. References of previous related reviews were screened to identify potential studies. Additionally, only studies that were written or translated into the English language were included in the analysis. To be considered for inclusion, studies had to report on 1 or more of the outcomes of interest. Studies involving nerve grafting or transfer procedures were excluded from the analysis.

### Methodological quality of the included studies

To evaluate the quality of the articles included in this analysis, the Joanna Briggs Institute Critical Appraisal Tool was used.^7^ This tool assesses the quality of cohort studies based on various factors, including the appropriateness of the sample frame, sampling method, sample size adequacy, data analysis, methods for identifying and measuring the relevant condition, statistical analysis and adequacy of response rate.

### Data extraction

From the studies that met the inclusion criteria, detailed information such as study and intervention characteristics were extracted, along with the outcome data. To review the characteristics of the studies, the following information was extracted: the country where the study was conducted, size and intervention, comparison group if present, outcome measures and study design.

### Statistical analysis

Statistical heterogeneity was assessed via the I^2^ and Cochran's Q test values, where I2 value of 25%, 50% and 75% represented low, moderate and high degrees of heterogeneity, respectively.^8^ A two-tailed P-value <0.05 was considered as the threshold for statistical significance.

For postoperative changes in smile excursion and the angle of the mouth while smiling, there was consistent data in the included studies and therefore a meta-analysis was conducted.

The statistical analyses and meta-analyses were conducted using Stata, version 14 (StataCorp, College, TX) and Cochrane Review Manager 5.3 (London, UK), following the DerSimonian and Laird random-effects model.^9^ A random-effects model was used for all analyses regardless of heterogeneity measures, as evidence has shown more robust effect estimates with random-effects models compared to fixed-effects models.^10^^,^^11^ For continuous variables, the postoperative mean difference (MD) in smile excursion and the angle of the mouth while smiling were calculated to obtain the pooled-effect estimates. If means and SDs were unavailable, we used the formula proposed by Cai et al. (https://smcgrath.shinyapps.io/estmeansd/) to estimate these values.^12^ The methodology was chosen in consensus by all participating authors. The senior author supervised the statistical analysis.

## Results

**Figure 1:**
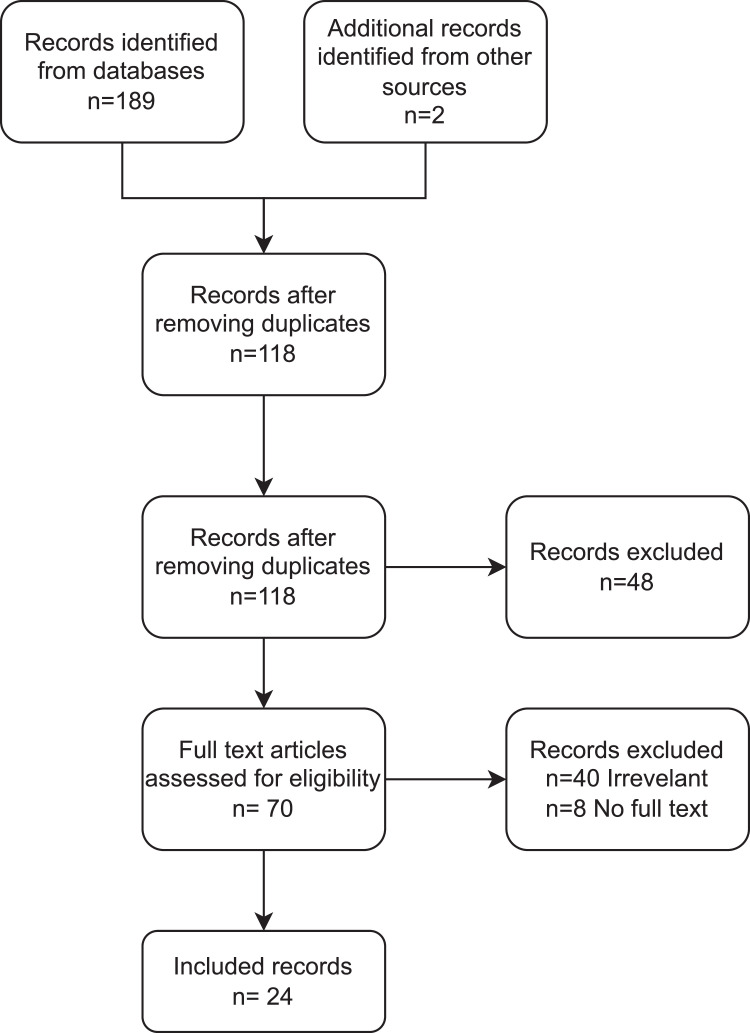
PRISMA diagram. PRISMA, preferred reporting items for systematic reviews and meta-analyses.

Figure 1 summarizes the search results, according to the preferred reporting items for systematic reviews and meta-analyses guidelines. A total of 189 studies were identified based on our search strategy and 71 duplicates were removed. In total, 48 more studies were excluded upon preliminary screening of the title and abstract. Finally, 70 full-text studies were assessed, resulting in the exclusion of 40 studies and 8 studies due to irrelevancy and the unavailability of the full-text, respectively. Two studies were added after examining the reference lists of the included studies. Thus, 24 studies (723 patients) fulfilled the inclusion criteria. Table 1 provides an overview of the characteristics of the included studies, including study design, country of origin, patients involved, methodology and outcome(s) measured. The risk of bias of the included studies was low (Table 2a, Table 2b).

**Table 1:** Summary of the included studies.

Study	Study Design	Duration	Compare	Follow-up	Method	n	Outcomes							
							1	2	3	4	5	6	7	8
Askar et al., 2020	Retrospective case series	January 2008-May 2017	-	-	A modified technique of temporalis muscle transposition technique (MTMT)	11	/							
Balaji 2002	Prospective case series	-	-	5 y	Temporalis transfer	5	/							
Brichacek et al., 2017	Retrospective case series	2003-2010	-	3.4 mo	Minimally invasive temporalis tendon transfer	25				/				
Briedahl et al., 1996	Prospective case series	-	-	-	Temporalis transfer	7	/							
Croise et al., 2019	Prospective case series	September 2017 and June 2018	-	6 mo	LTM	13					/			/
Foirest et al., 2017	Retrospective case series	November 2010-December 2013	-	-	LTM	25		/		/				
Gousheh et al., 2011	Retrospective cohort study	1987-2007	Two-stage gracilis, rectus abdominis, single-stage latissimus dorsi microneurovascular muscle transfers, cross-facial facial nerve neurotization procedures, neurotizations by the hypoglossal nerve, and neurotizations by the spinal accessory nerve	1-20 y	TTT, LTM	77	/							
Griffin et al., 2012	Retrospective cohort study	-	Irradiated patients	>3 mo	Temporalis tendon transfer	17		/	/					
Har-Shai et al., 2010	Prospective case series	-	-	3-12 mo	LTM	15	/							
Holtmann et al., 2017	Retrospective case series	2011-2015	-	-	Temporalis muscle transposition + facelift, nasal valve suspension, endoscopic brow lift, and eyelid reconstruction	20								/
House et al., 2021	Retrospective case series	April 2011-June 2018	-	11.4 mo	TTT, LTM	32		/	/					
Kecskes et al., 2009	Retrospective cohort study	1998-2005	Hypoglossal-facial nerve coaptation		LTM	10				/				/
Kwon et al., 2020	Retrospective case series	2017-2019	-	-	Temporalis muscle tendonperiosteum (T-P) compound surgical method	6			/					
Martineau et al., 2022	hybrid cross-sectional and retrospective cohort study	-	-	-	LTM (the only major differences are that the zygomatic arch is left intact and not osteotomized, and that the coronoid process osteotomy is performed through the nasolabial incision)	15			/			/		/
Nguyen et al., 2020	Retrospective cohort study	2008-2016	CFNG-FGMT	1 mo-25 y	LTM	6		/					/	
Oyer et al., 2018	Retrospective cohort study	1 July 2010-30 July 2014	Gracilis free muscle transfer	-	Temporalis tendon transfer	14		/	/					
Ozturan et al., 2015	Retrospective case series	-	-	-	LTM	4							/	
Panciera et al., 2017	Retrospective case series	2011-2016	-	6 y	LTM V1 and V2	11		/	/					/
Renault et al., 2017	Retrospective case series	January 2000-January 2013	-	-	Temporalis tendon transposition	7							/	
Sidle et al., 2011	Retrospective case series	-	-	-	Orthodromic temporalis transfer	10								/
Silan et al., 2020	Retrospective case series	March 2015-September 2018	-	21.5 mo	A modification of the orthodromic temporalis transposition using a cryopreserved fascia lata allograft	7								/
van Veen et al., 2018	Retrospective case series	1993-2017	-	>6 mo	Modified Rubin temporalis transposition (MRTT)	23				/		/		
van Veen et al., 2018	Cross-sectional cohort study	-	Gracilis transplantation	6.2 y	Temporalis muscle transpositions	12		/	/			/		/
Veyssière et al., 2015	Retrospective cohort study	-	-	7.3-9.3 mo	LTM	34	/

**Table 2a:** Methodological quality of the included cohort studies using the Joanna Briggs Institute (JBI) Critical Appraisal Tool

Study	Title	Q1	Q2	Q3	Q4	Q5	Q6	Q7	Q8	Q9	Q10	Q11	Total score
Gousheh et al., 2011	Treatment of facial paralysis: dynamic reanimation of spontaneous facial expression-apropos of 655 patients	/	/	/	/	/	/	/	/	/	/	/	11
Griffin et al., 2012	Outcomes following temporalis tendon transfer in irradiated patients	/	/	/	/	/	/	/	/			/	9
Kecskes et al., 2009	Lengthening temporalis myoplasty versus hypoglossal-facial nerve coaptation in the surgical rehabilitation of facial palsy: evaluation by medical and nonmedical juries and patient-assessed quality of life	/	/	/	/	/	/	/	/			/	9
Martineau et al., 2022	Public and patients' perceptions of facial reanimation using lengthening temporalis myoplasty	/	/	/	/	/	/	/	/			/	9
Nguyen et al., 2020	Comparison of Lengthening Temporalis Myoplasty and Free-Gracilis Muscle Transfer for Facial Reanimation in Children	/	/	/	/	/	/	/	/			/	9
Oyer et al., 2018	Comparison of Objective Outcomes in Dynamic Lower Facial Reanimation with Temporalis Tendon and Gracilis Free Muscle Transfer	/	/	/	/	/	/	/	/	/		/	10
van Veen et al., 2018	Gracilis transplantation and temporalis transposition in longstanding facial palsy in adults: Patient-reported and aesthetic outcomes	/	/	/	/	/	/	/	/			/	9
Veyssière et al., 2015	Lengthening temporalis myoplasty and facial paralysis from birth	/	/	/	/	/	/	/	/			/	9

Q1 1. Were the two groups similar and recruited from the same population?

Q2 2. Were the exposures measured similarly to assign people to the exposed and unexposed groups?

Q3 3. Was the exposure measured in a valid and reliable way?

Q4 4. Were the confounding factors identified?

Q5 5. Were the strategies to deal with confounding factors stated?

Q6 6. Were the groups/participants free of the outcome at the start of the study (or during exposure)?

Q7 7. Were the outcomes measured in a valid and reliable way?

Q8 8. Was the follow-up time reported and sufficient to be long enough for outcomes to occur?

Q9 9. Was the follow-up complete, and if not, were the reasons for the loss to follow-up described and explored?

Q10 10. Were the strategies to address the incomplete follow-up used?

Q11 11. Was the appropriate statistical analysis used?

**Table 2b:** Methodological quality of included case series using Joanna Briggs Institute (JBI) Critical Appraisal Tool

Study	Title	Q1	Q2	Q3	Q4	Q5	Q6	Q7	Q8	Q9	Q10	Total score
Askar et al., 2020	A Modified Technique of Transposition of Temporalis Muscle in Selected Cases of Longstanding Facial Paralysis	/	/	/	/	/	/	/	/	/		10
Balaji 2002	A modified temporalis transfer in facial reanimation	/	/	/	/	/	/	/	/			9
Brichacek et al., 2017	Objective Outcomes of Minimally Invasive Temporalis Tendon Transfer for Prolonged Complete Facial Paralysis	/	/	/	/	/	/	/	/		/	9
Briedahl et al., 1996	A modified surgical technique for temporalis transfer	/	/	/	/	/	/	/		/		8
Croise et al., 2019	Lengthening temporalis myoplasty and reduction of the swallowing oral phase dysfunction in facial palsy patients	/	/	/	/	/	/	/	/	/	/	9
Foirest et al., 2017	Smile Reanimation after Unilateral Facial Palsy by Lengthening Temporalis Myoplasty: Objective and Subjective Evaluation on 25 Cases	/	/	/		/	/	/	/	/	/	9
Har-Shai et al., 2010	Intraoperative muscle electrical stimulation for accurate positioning of the temporalis muscle tendon during dynamic, one-stage lengthening temporalis myoplasty for facial and lip reanimation	/	/	/	/	/	/	/	/			8
Holtmann et al., 2017	Outcome of a graduated minimally invasive facial reanimation in patients with facial paralysis	/	/	/		/	/	/	/	/	/	9
Kwon et al., 2020	Modified temporalis tendon transfer extended with periosteum for facial paralysis patients	/	/	/		/	/	/	/	/	/	9
Ozturan et al., 2015	Electromyographic Evaluation of Temporalis Muscle Following Temporalis Tendon Transfer (Facial Reanimation) Surgery	/	/	/		/	/	/	/	/		10
Panciera et al., 2017	Lengthening Temporalis Myoplasty: Objective Outcomes and Site-Specific Quality-of-Life Assessment	/	/	/	/	/	/	/	/	/	/	9
Renault et al., 2017	Electromyographic assessment of the temporalis muscle prior to a lengthening myoplasty in patients with Moebius syndrome	/	/	/		/	/	/	/	/	/	8
Sidle et al., 2011	Modification of the orthodromic temporalis tendon transfer technique for reanimation of the paralyzed face	/	/	/	/	/	/	/	/			9
Silan et al., 2020	Cryopreserved fascia lata allograft use in surgical facial reanimation: a retrospective study of seven cases	/	/	/		/	/	/	/	/		8
van Veen et al., 2018	Keeping the fat on the right spot prevents contour deformity in temporalis muscle transposition	/	/	/	/	/	/	/	/		/	8
House et al., 2021	Temporalis Tendon Transfer/Lengthening Temporalis Myoplasty for Midfacial Static and Dynamic Reanimation After Head and Neck Oncologic Surgery	/	/	/	/	/	/	/	/	/	/	10

Q1 Were there clear criteria for inclusion in the case series?

Q2 Was the condition measured in a standard, reliable way for all participants included in the case series?

Q3 Were valid methods used for identification of the condition for all participants included in the case series?

Q4 Did the case series have consecutive inclusion of participants?

Q5 Did the case series have complete inclusion of participants?

Q6 Was there clear reporting of the demographics of the participants in the study?

Q7 Was there clear reporting of clinical information of the participants?

Q8 Were the outcomes or follow-up results of cases clearly reported?

Q9 Was there clear reporting of the presenting site(s)/clinic(s) demographic information?

Q10 Was statistical analysis appropriate?

### Existing technique and modifications in the included studies

Overall, among the included studies, 9 (38%) studies described LTM based on the technique described by Labbé et al. and its modifications, and 13 (54%) studies discussed TTT based on the technique described by McLaughlin et al. and Rubin et al. with different variations. Two (8%) studies assessed both techniques.^13^^,^^14^ LTM, also known as Labbé procedure, separates the temporalis muscle from the temporal fossa and allows lengthening by redistribution of the muscular fibers to the detriment of the posterior third which allows the transfer of the coronoid tendinous insertions onto the lips for lip reanimation.^15^ TTT involves the transfer of a segment of the temporalis muscle, which is innervated by the deep temporal nerves arising from the mandibular division of the trigeminal nerve, to the corner of the mouth to restore the dynamic function of the orbicularis oris muscle.

### Spontaneous Smile

Six studies were found to evaluate spontaneous or emotional smile outcomes and the results were conflicting. In the studies by Har-Shai et al. and Veyssière et al., most patients (n = 49) who underwent LTM or TTT were able to achieve a satisfactory, truly spontaneous smile in 3 to 9 months.^16^^,^^17^ However, in the other 4 studies, none of the patients (n = 100) were able to achieve spontaneous smile.^18^^,^^19^^,^^20^^,^^21^ Nevertheless, the definition of spontaneous remains largely subjective, heterogeneous and ill-defined across all 6 studies. Only 1 study by Veyssière et al. clearly defined the spontaneity as smile with contraction of the transposed temporal muscle (temporal smile), without masseter contraction.

### Postoperative Changes in Smile Excursion

**Figure 2:**
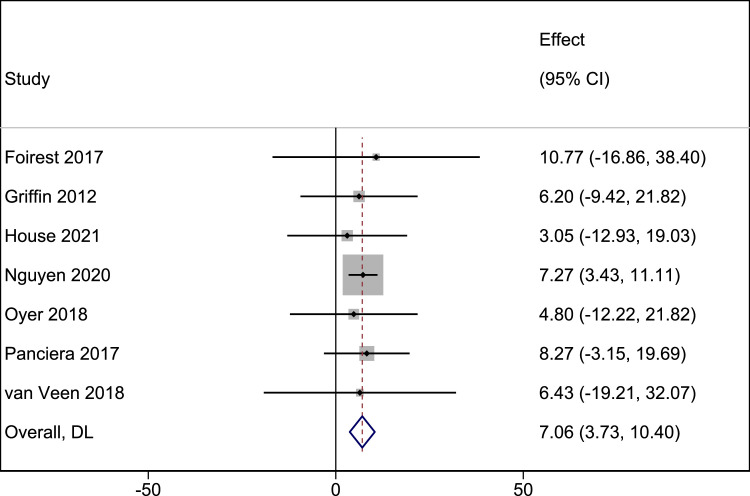
Postoperative changes in smile excursion.

Seven studies (82 patients) were included in the meta-analysis of changes in smile excursion postsurgery defined as the distance from the midline lower lip to the oral commissure.^22^, ^23^, ^24^, ^25^, ^26^, ^27^, ^28^ The pooled MD in smile excursion postsurgery was 7.06 mm (95% CI: 3.73-10.40, P < 0.001; I^2^ = 0%) (Figure 2).

### Postoperative Changes in the Angle of the Mouth while smiling

**Figure 3:**
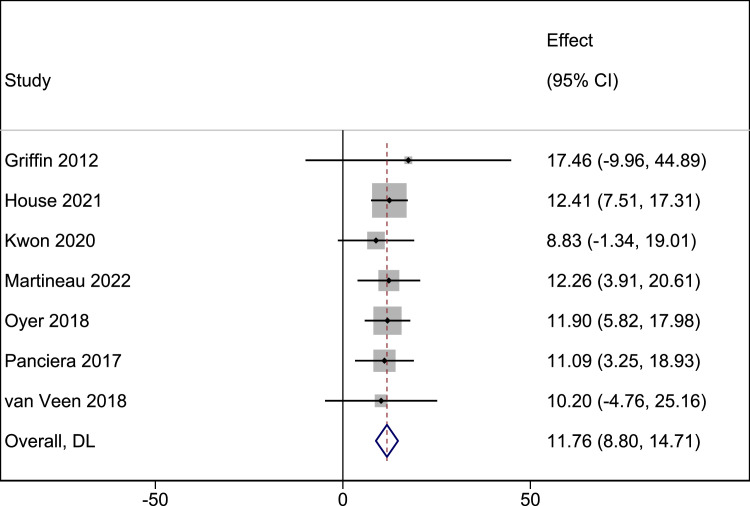
Postoperative changes in the angle of the mouth while smiling.

Seven studies (107 patients) were included in the meta-analysis of changes in the angle of the mouth while smiling postsurgery.^23^^,^^24^^,^^26^, ^27^, ^28^, ^29^, ^30^ The pooled MD changes in the angle of the mouth while smiling postsurgery was 11.76° (95% CI: 8.80-14.71, P < 0.001; I^2^ = 0%) (Figure 3).

### Validated Rating Scale Of Symmetry

Validated rating scales were used in 4 studies to assess the outcomes of LTM/TTT. Terzis and Noah grade, a combined aesthetic and functional scale ranging from 1 (poor) to 5 (excellent) based on standard photographic analysis was used in 2 studies. In the first study, postoperative improvement was noted using the Terzis scoring system with the scores being mostly in consensus among the expert with no patients achieving a worsened outcome.^31^ Similarly, in another study, 58% of the patients were evaluated as excellent or good by expert and nonexpert observers at intermediate smile state after LTM.^22^ For patients at the intermediate and maximum smile states combined, 88% of the patients were evaluated as improved in the postoperative photographs, by both groups, experts and nonexperts according to the Terzis scoring system. No result was evaluated as poor in this series. In a study by van Veen et al., most patient scored “fair”/“good” on the May classification (a subjective rating scale emphasizing on symmetry and teething showing in smiles ranging from excellent, good, fair to poor) despite a predominance of “poor” scores before TTT surgery.^32^ In another study evaluating the outcomes of facial reanimation surgeries using rating scales including the House-Brackmann, Sunnybrook, Yanagihara and Freyss by both medical juries, a statistically superior outcome was observed for hypoglossal-facial coaptation compared to LTM across all 4-rating scale, regardless of the types of the coaptation.^33^

### Functional Outcomes

The prospective study by Croise et al. found that lip continence improved steadily over time after surgery, with statistically significant differences at 3 and 6 months after surgery.^34^ Drooling also decreased steadily after surgery, with a significant difference at 6 months, measured by Drooling Severity and Frequency Scale. Bolus residue at the end of mastication decreased steadily over time, with significant differences at 3 and 6 months after surgery but mastication duration did not show significant differences over time.

### Aesthetic outcomes

Three studies investigated the aesthetic outcomes post-LTM/TTT. The first study found that the public's perception of facial appearance improved after LTM, with a significant decrease in disfigured, important to repair, bothersome, severity score which measures facial perception of patients and improvement in attractiveness score.^30^ The second study found no significant difference in the aesthetic outcomes between the gracilis free functional muscle transfer (FFMT) and temporalis muscle transpositions, as rated by laypersons.^28^ In a study evaluating temporal hollowing and zygomatic bulging using 3D photograph of 8 patients, it was found that the volume of temporal asymmetry was not significantly different (1.3 ml vs 0.6 ml) but significant difference was found in zygomatic bulging (3.3 ml vs 1.8 ml) compared to the control group.^32^

### Electromyography outcomes

Three studies reported the use of surface electromyography of the temporalis muscle (sEMG) for patients who underwent LTM/TTT. Two studies assessed the association between sEMG reading and postoperative outcomes. One of the studies which reviewed 18 patients retrospectively found that patients with normal preoperative EMG results had better postoperative outcomes than those with abnormal results.^35^ The other study demonstrated unhampered EMG activity of the temporalis muscle on the operated side and over-correction could lead to a lower reading for the temporalis and masseter muscles.^36^ The study by Nguyen et al. showed a significant increase in sEMG muscle recruitment in the LTM and CFNG-FGMT groups 3 months postoperatively with a steady rate of increase over time while higher muscle recruitment per month was recorded in the LTM group.^25^

### Patient-reported outcomes

Eight studies investigated the patient-reported outcome measures (PROMs) for patients post-LTM and TTT. The reported measures included the Glasgow Benefit inventory (GBI), facial disability index (FDI) scores, facial clinimetric evaluation scale (FaCE) questionnaire and Deglutition Handicap Index (DHI).

### Glasgow Benefit inventory

GBI was employed in 2 studies and both studies reported positive outcomes. The GBI questionnaire comprised 18 health questions assessing changes in health, with responses scored on a 5-point Likert scale, ranging from a large deterioration to a substantial improvement in health status. The questions assess the general perception of wellbeing, with psychological, social and physical subscale. Score ranging from −100 (maximal harm) through 0 (no change) to 100 (maximal benefit). In a study by Holtmann et al., the total GBI and general subscale scores were positive, indicating a statistically significant benefit for patients who underwent temporalis muscle transposition.^37^ Higher GBI scores were associated with patients who felt better about themselves after surgery and were less inconvenienced by their health problem. However, there was no significant impact on social support and general physical health. Similarly, in another study, the GBI results showed a net improvement regardless of the procedure (LTM, hypoglossal-facial coaptation), and patients reported significant improvements in their daily and professional lives.^33^ Most patients were satisfied with the surgery, would consent to it again and found it useful.

### FDI scores

Three studies reported the results of the FDI score after surgery. The FDI includes 10 questions regarding physical and social activities such as the ability to brush teeth, eat and speak; additional questions enquire about cornea protection, isolation, irritability, social activity and sleeping disorder. A significant improvement in self-perceived quality of life post-LTM was demonstrated in a retrospective review of 11 patients, with a significant increase in FDI score from 33.4 to 49.9 points.^27^ Meanwhile, the study by Silan et al. also showed an improvement in physical and social subscales of the FDI questionnaire in 6 out of 7 patients despite the complications after partial temporalis tendon transfer with the use of cryopreserved fascia lata allograft, with only 1 patient reporting an improvement in the physical subscale but a worsening in the social subscale.^38^ In a retrospective cohort study, it was reported that all 10 patients underwent LTM. Although they were less disabled after surgery, they still had some complaints, with a mean FDI score of 56/100 for the physical portion and 69/100 for the social portion. The difference in FDI scores was not significant compared to 48 hypoglossal-facial coaptation patients.^33^

### FaCE questionnaire

Three studies employed the FaCE questionnaire which is a validated and commonly used questionnaire to evaluate disease-specific quality of life in facial palsy. In the study by Sidle et al., 10 consecutive patients who underwent orthodromic transfer of the temporalis tendon were retrospectively reviewed and FaCE questionnaire score significantly increased threefold postsurgery.^39^ Similarly, in another study involving 15 patients undergoing LTM, significant improvement was observed in the FaCE-F questionnaire (a Canadian French version) score, particularly in the subsection of oral function and facial movement.^30^ Interestingly, in the study comparing 10 patients who had FFMT and 12 patients who had temporalis muscle transposition, slightly higher median oral function score was observed in the temporalis transposition group but none of the difference between 2 groups across total and sub scores were significantly different.^28^

### Deglutition handicap index

One study measured 3 aspects of disability evaluation (physical, functional and emotional/social) for 13 LTM patients using the DHI questionnaire.

The results showed that the mean overall DHI score was reduced after surgery, but the changes were not statistically significant at 3 or 6 months after surgery. However, there was a significant improvement in physical handicap score at both time points, while the changes in functional and social disabilities were not significant.^34^

## Discussion

This study presents a systematic review and meta-analysis of temporalis-based facial reanimation surgery, which offers promising results. The results indicate that temporalis-based facial reanimation surgery can significantly improve smile excursion and the angle of the mouth while smiling, while positively impacting aesthetic and functional outcomes and PROMs. Nevertheless, the study highlights the uncertainty surrounding the restoration of spontaneous smile in patients with facial paralysis. Overall, the temporalis-based facial reanimation surgery is a promising option for addressing the negative effects of facial nerve paralysis on the patients’ quality of life.

Normally facial weakness in patients with facial nerve paralysis mostly encompasses the middle and lower third of the face. These include patients with the chronic sequelae of facial nerve paralysis and in most patients there is an absence of functional facial muscles. Facial reanimation surgery in the patients requires a two-step approach.^4^ The initial step consists of cross-facial nerve grafting, mostly followed by micro-neurovascular muscle transfer. For patients with some remaining functional facial musculature, other options such as the masseter or hypoglossal jump procedures can be good options.^4^ Temporalis-based facial reanimation surgery is a promising alternative that preserves the motor innervation to the temporalis muscle and can result in dynamic correction of facial paralysis. This technique offers several benefits, including technical simplicity, a single-step approach, low complication rates and reduced operative duration. Owing to the insufficient evidence of its superior effectiveness over other techniques and its relative rarity in practice, some surgeons may be hesitant to use temporalis-based facial reanimation surgery. Temporalis-based facial reanimation surgery is generally considered a second-line option for patients who are not appropriate candidates for microvascular free muscle transfer or those who choose not to undergo this procedure; therefore, masseter muscle transfer is usually recommended as an ancillary procedure. This study demonstrated an optimal result on smile excursion which is comparable to other techniques.

The achievement of spontaneous and emotional smiles through nerve grafting is well-established, highlighting their emotional nature rather than being a voluntary effort.^40^^,^^41^ However, it remains unclear whether the brain plasticity induced by temporalis-based facial reanimation surgery is sufficient to generate spontaneous smiles. In our study, we observed highly polarized results concerning spontaneous smiles despite Garmi et al.’s study indicating that LTM can result in cortical reorganization, contributing to smiling and chewing action of the temporalis muscle.^42^ Our findings suggest that the current data on the subject are conflicting, and additional research is required to clarify the role of LTM in inducing spontaneous smiles. Furthermore, the lack of a standardized definition of spontaneous and emotional smiles has resulted in difficulties in quantifying the outcomes of various treatments, emphasizing the need for uniform measurement standards to ensure data reliability.

PROMs play a crucial role in evaluating the success of facial paralysis treatments, including the temporalis-based facial reanimation surgery. The use of PROMs allows for the measurement of a patient's health status, functional status and quality of life based on their own perceptions and experiences, which is particularly important when traditional clinical measures may not fully capture the effects of facial paralysis. Among the various tools used to measure the outcomes in facial paralysis patients, the FaCE questionnaire and FDI have become the most widely used owing to their patient population specificity. The 2 tools under investigation exhibited a similar level of reliability, and neither one appeared to exhibit unequivocal superiority over the other.^43^^,^^44^ The association between aesthetic and functional outcomes with the patient's quality of life remained unclear and further research is necessary to fully understand its potential.

The use of sEMG also shows potential as a valuable adjunct for guiding patient selection and monitoring temporalis muscle function. The utilization of EMG has been documented in various types of facial reanimation surgeries as a means of prognosticating surgical outcomes or monitoring them.^45^^,^^46^ The procedure's inherent noninvasive nature renders it feasible for implementation in all age groups.

A major difficulty with examining the outcomes of interest in facial reanimation surgery was encountered owing to the many ways in which surgical results were evaluated. Most studies used observer dependent surveys to evaluate the outcomes. These methods are subject to the possibility of self-serving bias. This also makes comparison with other surgical procedures difficult because of inhomogeneous reporting of patient selection criteria and nonuniformity in outcome measurements. Limited data are available to compare temporalis-based facial reanimation surgery with other modalities. Only 5 out of 24 included studies comparatively analyzed the temporalis-based facial reanimation surgery with other types of facial reanimation surgery. The wide variety of outcome variables in the literature posed substantial restrictions in the current systematic review of the existing literature and prohibited us from performing meta-analyses. The outcomes of the facial reanimation procedures may be impacted by the variations in surgical techniques and differences in facial rehabilitation protocols. Quantification of objective outcomes was inconsistent and posed another challenge because of the lack of a standardized outcome scoring system for facial reanimation. To propel the field of facial reanimation forward, it is vital to conduct outcome comparisons in a standardized manner. To accomplish this, an assessment system should possess the following qualities: simplicity, objectivity and reproducibility; robust inter- and intra-rater correlation and the ability to evaluate surgical outcomes in a clinical setting while also being suitable for research purposes. The absence of randomized controlled trials resulted in a dearth of robust evidence. To address this limitation, multicenter studies that incorporated standardized surgical techniques, measurement scales and postoperative physical therapy regimens should be conducted, with a minimum follow-up period of 1 year.

## Conclusion

Overall, temporalis-based facial reanimation surgery holds promise in alleviating the detrimental impact of facial nerve paralysis on the patients’ quality of life. However, this study underscores the existing uncertainties surrounding the technique and emphasizes the necessity for additional research. Furthermore, the comparative analysis with other techniques remains unclear, thus warranting the implementation of multicenter studies with well-defined methodologies.

## Author contributions

**Initial idea:** SP

**Literature search and data analysis:** ZW, SP

**Writing the article:** ZW, FdJ, KI, NvH, SP

**Final approval:** ZW, FdJ, KI, NvH, SP
